# New Clues to Prognostic Biomarkers of Four Hematological Malignancies

**DOI:** 10.7150/jca.69274

**Published:** 2022-05-09

**Authors:** Samson Pandam Salifu, Albert Doughan

**Affiliations:** 1 Department of Biochemistry and Biotechnology, Kwame Nkrumah University of Science and Technology (KNUST), Kumasi, Ghana.; 2 Kumasi Centre for Collaborative Research in Tropical Medicine (KCCR), Kumasi, Ghana.

**Keywords:** Hematologic malignancies, Hub genes, RNA-seq, Lymphoma, Biomarker

## Abstract

Globally, one out of every two reported cases of hematologic malignancies (HMs) results in death. Each year approximately 1.24 million cases of HMs are recorded, of which 58% become fatal. Early detection remains critical in the management and treatment of HMs. However, this is thwarted by the inadequate number of reliable biomarkers. In this study, we mined public databases for RNA-seq data on four common HMs intending to identify novel biomarkers that could serve as HM management and treatment targets. A standard RNA-seq analysis pipeline was strictly adhered to in identifying differentially expressed genes (DEGs) with DESeq2, limma+voom and edgeR. We further performed gene enrichment analysis, protein-protein interaction (PPI) network analysis, survival analysis and tumor immune infiltration level detection on the genes using G:Profiler, Cytoscape and STRING, GEPIA tool and TIMER, respectively. A total of 2,136 highly-ranked DEGs were identified in HM vs. non-HM samples. Gene ontology and pathway enrichment analyses revealed the DEGs to be mainly enriched in steroid biosynthesis (5.075×10^-4^), cholesterol biosynthesis (2.525×10^-8^), protein binding (3.308×10^-18^), catalytic activity (2.158×10^-10^) and biogenesis (5.929×10^-8^). The PPI network resulted in 60 hub genes which were verified with data from TCGA, MET500, CPTAC and GTEx projects. Survival analyses with clinical data from TCGA showed that high expression of *SRSF1*, *SRSF6*, *UBE2Z* and *PCF11,* and low expression of* HECW2* were correlated with poor prognosis in HMs. In summary, our study unraveled essential genes that could serve as potential biomarkers for prognosis and may serve as drug targets for HM management.

## Background

Hematological malignancies (HMs) present a global health burden worsened by a lack of precise diagnostic, treatment and prognostic biomarkers. An estimated 1.24 million cases of HMs are diagnosed yearly across the globe, accounting for about 6% of all cancer cases 1. As of 2020, HMs case fatality rate stood at 58% and culminated in approximately 7% of all cancer deaths worldwide 1. This is an improvement to the statistics recorded in 2017, where HMs constituted 8.6% of all cancer cases and 11.5% of all cancer deaths worldwide 2. However, there was no corresponding reduction in the case-fatality rate from 2017 (52%) to 2010 (58%). Al-Azri 3 attributed the overall poor survival of HM patients to late diagnosis.

Recent advances in cancer therapies such as immunotherapy, stem cell transplantation, gene therapy and chemotherapy have improved HM cancers treatment. However, early detection continues to be a challenge. For screening and identification of HMs, ranges of assays are available such as blood tests, imaging (CT, X-ray or PET scans) tests and bone marrow biopsies. However, each of these methods has its drawbacks, including (1) false negative or positive results, (2) overdiagnosis of cases that could lead to unnecessary treatment and psychological stress 4 and (3) exertion of unnecessary worry and risk on a patient who may not have HM. For these reasons, it is critical to discover novel diagnostic and prognostic biomarkers that will be effective in HM diagnosis.

Liquid biopsies have recently supplanted traditional tissues biopsies as the preferred choice of diagnosis of HMs 5, 6. It provides a less painful, less invasive and increases the testing rate of HMs. Unfortunately, liquid biopsies can only detect circulating tumor cells (CTCs) and cell-free DNA (cfDNA), which may be present in low concentrations in the patient's blood and the tests may not be sensitive enough to detect them 6. This necessitates the need for more sensitive, accurate and reliable biomarkers for HM diagnosis.

In recent years, the introduction of inhibitors targeting immunological checkpoints such as PD-1/PD-L1 and CTLA-4 has resulted in significant paradigm shifts in treating hematological malignancies 7. Recent findings indicate that checkpoint inhibition appears to be a promising treatment option for certain types of hematologic malignancies 8. However, the use of checkpoint inhibitors is accompanied by significant side effects and high costs, and only a small percentage of patients appear to benefit clinically 9. This highlights the critical need for biomarkers to identify patients more likely to respond to treatment and/or experience fewer adverse effects. To this end, there have been reports on biomarkers that can serve as a diagnostic, prognostic and therapeutic target for HM management. Popular among these include the Cluster of Differentiation 47 (CD47) 10, 11, CD123 12 and miR-155 13. Although several antagonists of CD47, CD123 and miR-155 have been studied *in vitro* and *in vivo* with promising results using cell lines and mouse models of hematological malignancy, these studies focused on a specific HM at a time. Our approach leverages this limitation by considering hematological malignancies as a unity in identifying potential biomarkers to diagnosis and prognosis.

Multiple HMs may have similar gene expression profiles that could promote tumor progression 14. However, these genes have not been fully explored. Analyzing the transcriptomes of multiple HMs simultaneously will be vital in identifying the genes that HMs share in common, which will further enable the elucidation of their common signaling pathways that promote oncogenesis. These could be applied in the development of therapeutics and diagnostics to manage HMs effectively. In the present study, we contributed to the existing pool of HM biomarkers by identifying novel genes unique to HM patients that could serve as potential diagnostic and prognostic targets for HM treatment and management.

## Materials and methods

### Data sources

In this study, we mined public databases for RNA-seq data on chronic lymphocytic leukemia (CLL), acute myeloid leukemia (AML), acute lymphocytic leukemia (ALL) and Burkitt lymphoma (BL). We settled on four datasets generated by Cocciardi *et al*. 15 (AML), Black *et al*. 16 (ALL), Lombardo *et al*. 17 (BL) and CNAG-CRG 18 (CLL), based on our set inclusion criteria of at least ten samples, data being published within the last five years and cancer diagnosis being performed by at least two experienced oncologists. Table 1 provides a summary of the datasets used in this study. Ten paired-end FASTQ files were downloaded for each HM via NCBI-SRA. As a control group, we used mRNA data on lymphoblastoid cell lines (LCLs) from healthy non-cancer participants of the 1000 Genomes project. Our choice of data and control groups presents an unbiased representation of the various HMs.

### Quality control, trimming and mapping

FastQC 20 and MultiQC 21 were used for data quality assessment. Low-quality bases and adapter sequences were trimmed with Trimmomatic 22. Trimmomatic was also used to filter out reads, which were shorter than 20 bases pairs. Furthermore, the trimmed reads were aligned to the human reference genome (GRCh38) using the 2-pass mode of STAR aligner 23 under default parameters. Gene quantification was performed with featureCounts 24, with *gene_id* and* gene_biotype* attributes. A description of the tools used in this study has been provided in Table 2.

### Differential expression analysis (DEA)

**Figure 1:**
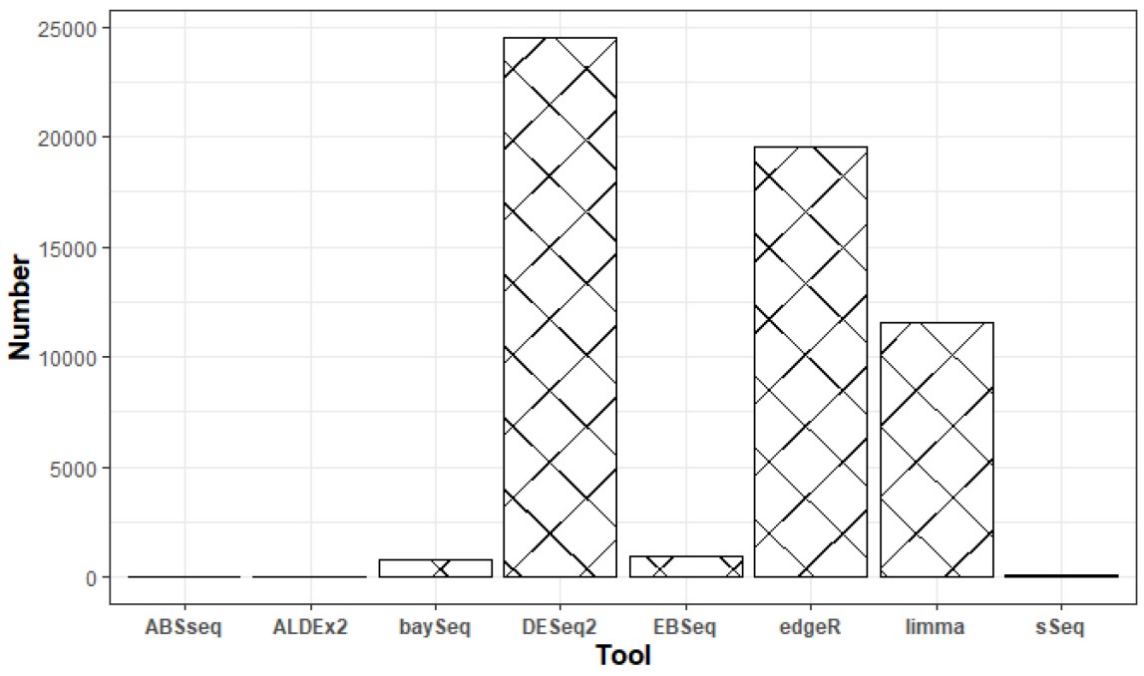
Google scholar citations for the respective DEA tools between January 2013 and March 2021.

We surveyed eight popular tools (ABSseq, ALDEx2, DESeq2, baySeq, EBSeq, edgeR, limma+voom and sSeq) used for differential expression analysis. Based on the total number of downloads and Google scholar citations (Figure 1), we settled on DESeq2, edgeR and limma+voom. We surmise that both the number of downloads and citations are commensurate to usage. Additionally, according to the tool's manual, all analyses were performed using default parameters following a step-by-step approach. Table 3 briefly describes the DEA tools used in this study.

### Gene ontology analyses

The overlapping set of genes identified by all the DEA tools were used for gene ontology analysis. The Database for Annotation, Visualization and Integrated Discovery (DAVID) 28 and G:Profiler's g:Gost 29 were used to identify the biological events and pathways for which the identified genes are involved in HMs. Adjusted P values (P_adj_) less than 0.05 were considered to be statistically significant, and all inferences were drawn from Functional Annotation Clusters with enrichment scores ≥ 1.3. Gene enrichment analysis using multiple databases provided corroborating evidence of the biological processes, molecular functions and biological pathways the genes are involved in HMs.

### Protein-protein interaction (PPI) network

Cytoscape 30, an open platform Bioinformatics program to visualize molecular interaction networks was used to visualize the protein-protein interaction (PPI) network of the genes. The STRING plugin 31 in Cytoscape was used to visualize the interactions between the common genes. PPIs with a confidence score of at least 0.9 were considered to be highly significant. Additionally, the Molecular Complex Detection (MCODE) 32 plugin in Cytoscape was used to identify the highly interconnected nodes (most closely associated genes) within the PPI network, which we termed hub genes.

### Hub genes expression in tumors

The Gene Expression Profiling Interactive Analysis (GEPIA) 33 online tool was used to analyze the expression of the hub genes in other human cancers. This was achieved through a systematic search across gene expression datasets from The Cancer Genome Atlas (TCGA) and Genotype-Tissue Expression (GTEx) projects.

### Hub-gene survival analysis

GEPIA tool was used to perform survival analysis on the hub genes. GEPIA employs data from TCGA and GTEx projects to perform analyses, including patient survival.

### Tumor immune infiltration levels

To investigate the association of gene expression patterns with tumor infiltration immune cells (TIIC), the Tumor Immune Estimation Resource (TIMER) web-based tool 34 was employed. Of the seven available TIMER modules (Gene, Survival, Mutation, SCNA, Diff Exp, Correlation, Estimation), we focused on SCNA to compare the tumor infiltration levels among hematologic malignancies with different copy number aberrations for a given gene. SCNA used the two-sided Wilcoxon rank-sum test to perform the analyses.

### Gene expression in different races, gender and age groups

Finally, we used the UALCAN web tool to explore the difference in hub gene expression in different age groups, races and gender. UALCAN uses cancer OMICs data from TCGA, MET500 35 and CPTAC 36 for biomarker identification and validation and explores the epigenetic regulation of gene expression. Table 4 describes all the web-based tools used in this study.

## Results

### Identification of differentially expressed genes (DEGs)

**Figure 2:**
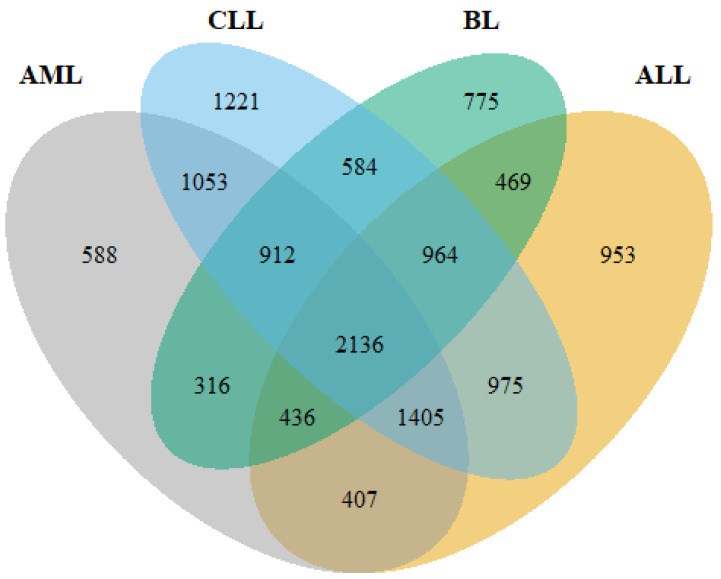
A Venn diagram showing the number of common genes among the four HMs (ALL, AML, CLL and BL).

Following pre-processing of the raw data, DEGs were identified using DESeq2, limma+voom and edgeR. Overall, 7745, 9250, 7253 and 6592 DEGs were obtained from ALL, CLL, AML and BL, respectively (Figure 2). The *intersect* function showed that 2,136 genes were common to all the HMs and served as the primary data for further analyses.

### Gene ontology (GO) analyses

GO and pathway enrichment analyses were performed using G:Profiler and DAVID to investigate the biological function of the shared DEGs. After removing all electronic GO terms, the results showed the DEGs to be significantly implicated in protein binding, catalytic activity and regulation of intracellular signal transduction. The most significant pathways were found to be steroid biosynthesis (P_adj_ = 5.075×10^-4^), cholesterol biosynthesis (P_adj_ = 2.525×10^-8^) and activation of gene expression by *SREBF* (P_adj_ = 1.617×10^-4^). Table 5 provides a detailed distribution of the top GO terms associated with the DEGs.

### PPI network and module selection

**Figure 3:**
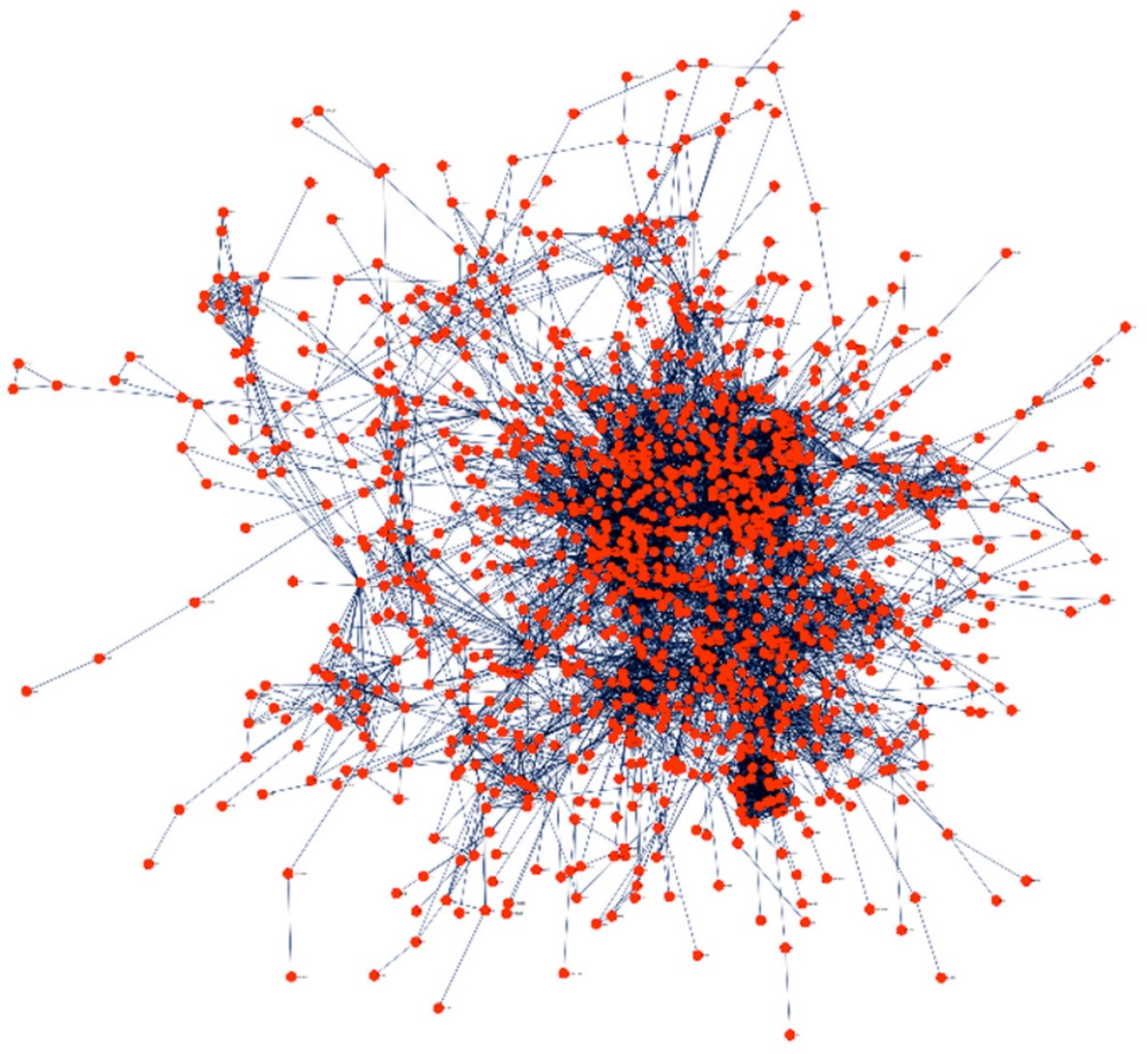
Protein-protein interaction network of the shared DEGs using STRING. The nodes and edges represent query DEGs and relationships between the DEGs, respectively.

PPI network was created to explore the relationships between proteins to study the molecular process of HMs in a systematic approach (Figure 3). The PPI network was developed using STRING through Cytoscape at a confidence score of > 0.9. Additionally, all singletons (nodes without any association) were excluded from further analyses. We observed that about 96% of the DEGs had a significant association with at least one other gene, confirming the agreement in DEG detection among the various datasets and analysis tools.

**Figure 4:**
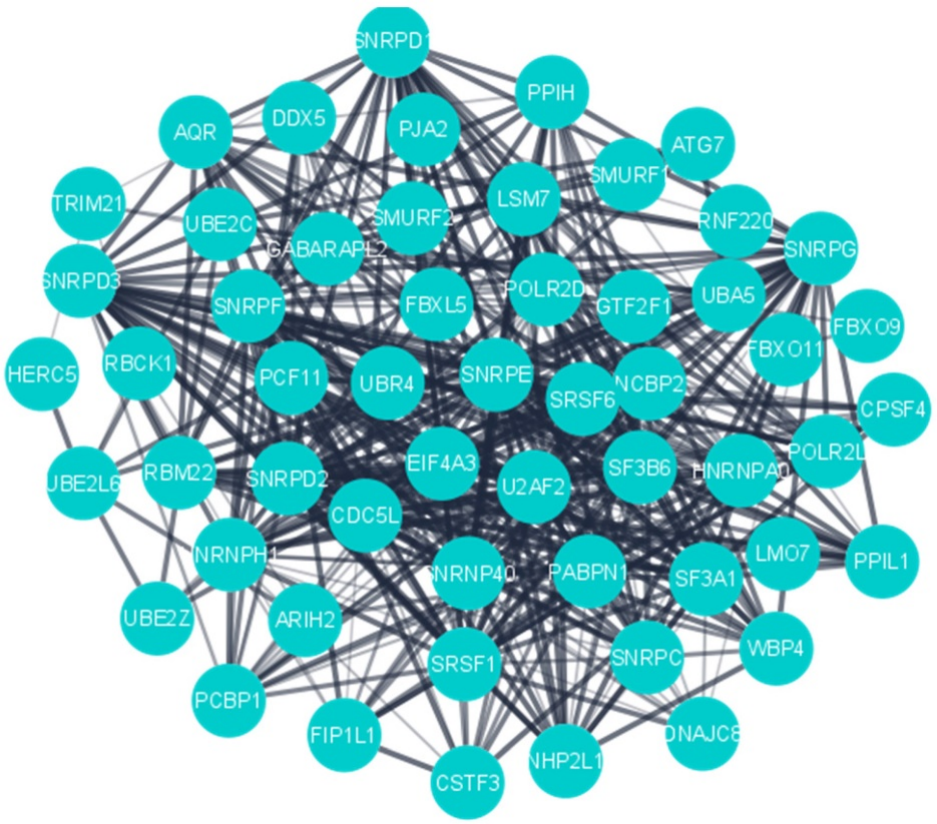
PPI network of the highly interconnected hub genes.

MCODE was used to detect the significant cluster modules present in the PPI network. It predicted 61 clusters and ranked them based on confidence scores (Figure 4). The module with the highest score (29.54) was selected and its genes (60) were used for enrichment analyses, which revealed ubiquitin-protein transferase activity (P_adj_ =3.78×10^-16^), ubiquitin-like protein transferase activity (P_adj_ = 1.08×10^-15^), mRNA splicing, via spliceosome (P_adj_ = 8.59×10^-36^), RNA splicing via transesterification reactions (P_adj_ = 1.13×10^-35^), mRNA processing (P_adj_ = 2.13×10^-32^) and mRNA metabolic process (P_adj_ = 1.11×10^-24^) to be most significant terms (**Supplementary Table S1**).

### Gene co-expression analysis

**Figure 5:**
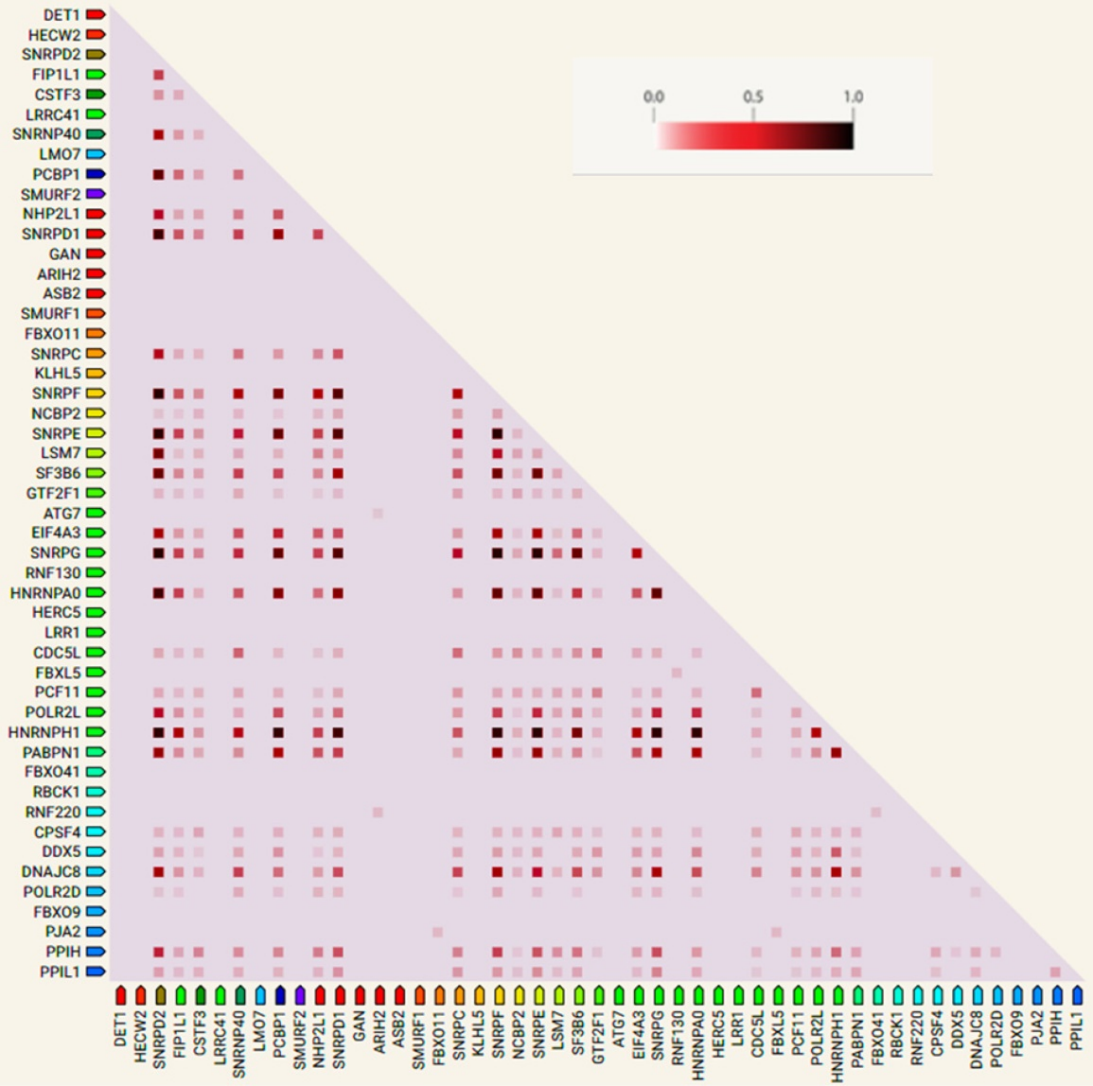
Co-expression analysis of the top 50 hub genes. Deeper colors depict stronger associations.

STRING was used to perform gene co-expression analysis to infer the interactions between the genes (Figure 5). The confidence scores used to generate the associations were obtained from RNA expression patterns and protein co-expression values from the ProteomeHD database. STRING could accommodate 50 genes out of the 60 hub genes; hence the last ten less significant genes were excluded. From Figure 5, *SNRPF*, *HNRNPH1*, *PABPN1*, *SNRPD2*, *SNRPE* and *SNRPG* positively interact with all the other genes in the cluster.

### Hub genes expression in hematologic malignancies and other cancers

**Figure 6:**
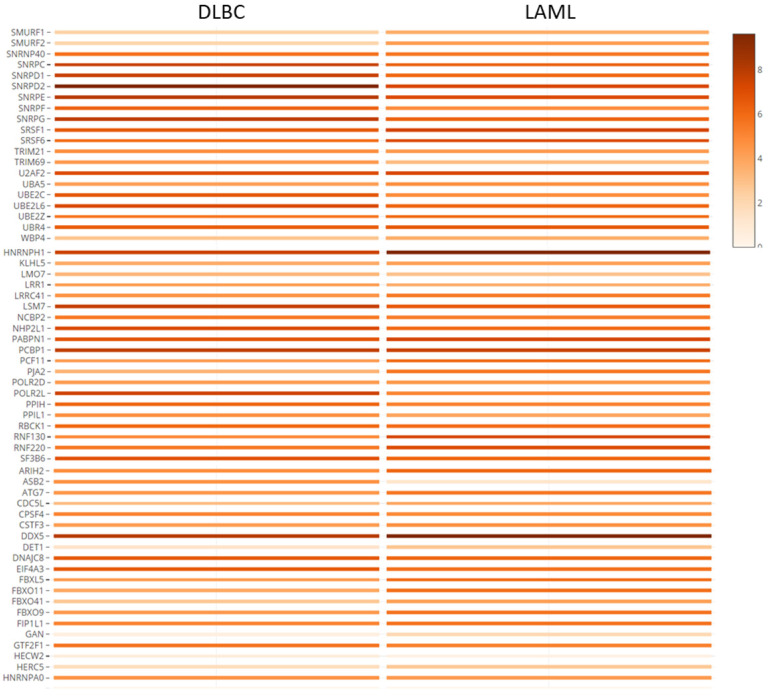
Co-expression analysis and verification of hub genes using TCGA and GTEx datasets. The shaded rectangles represent the median level of expression of a gene in DLBC and LAML. Color intensity is also proportional to expression levels.

The hub genes were verified with gene expression datasets from the TCGA and GTEx projects. Using GEPIA online tool, we explored the median expression levels of the hub genes in two hematologic malignancies (diffuse large B cell lymphoma (DLBC) and acute myeloid leukemia (LAML)). From Figure 6, we observed that most of the genes were highly expressed in the HMs under study. Importantly, *DDX5, HNRNPH, SNRPD2, PCBP1* and *SF3B6* showed very high expression levels in the LAML and DLBC. However, *ASB2* and *HECW2*; *DET1*, *GAN*, and* HERW2* were expressed minimally in the LAML and DLBC cancers, respectively.

**Figure 7:**
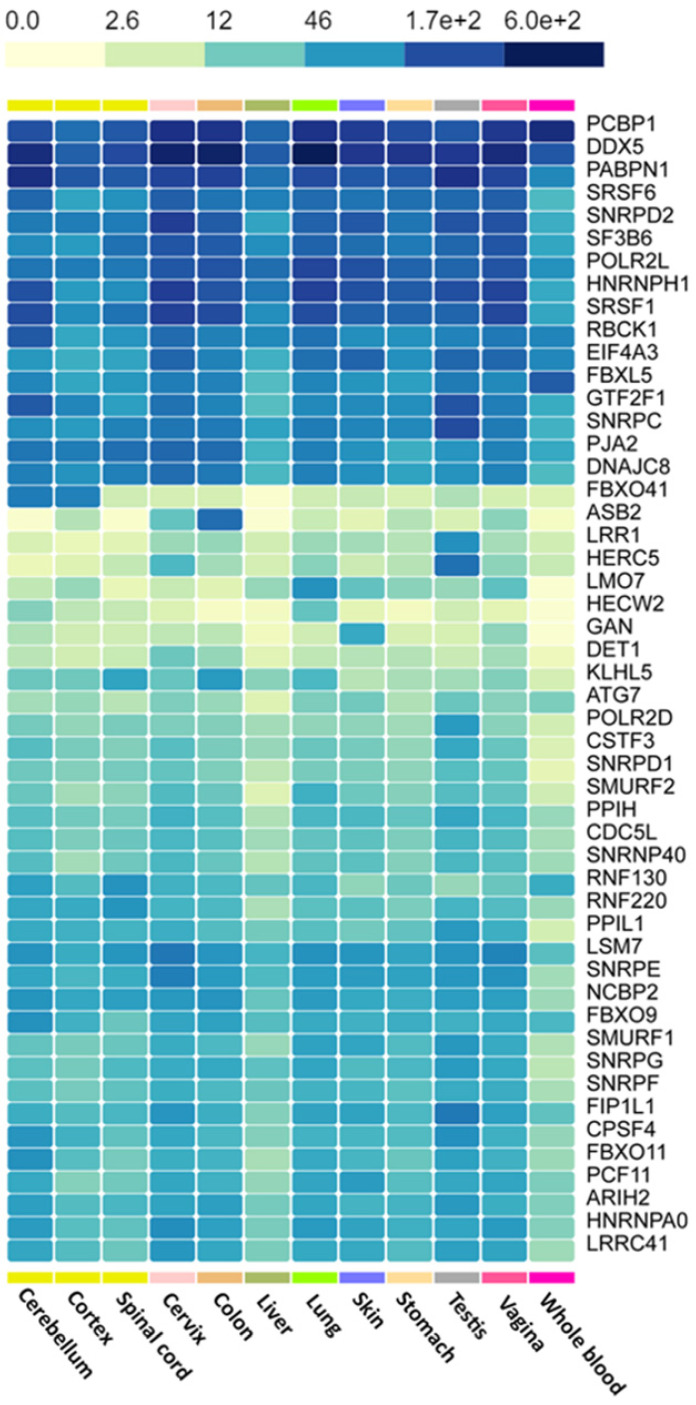
Heatmap of the expression of hub genes across HM-related and non-HM-related GTEx tissues. Color intensity is proportional to gene expression levels.

We used the GTEx portal to explore the level of hub gene expression in some tissues of the body (Figure 7). We focused on lymphocytes, blood cells, liver, spleen and brain as these are the organs most affected by hematologic malignancies 38. As a control group, we generated similar plots using tissues not directly affected by HMs, such as the vagina, cervix, testis and stomach. Comparing the gene expression levels (proportional to color intensity) for each gene, we found that all the highly expressed genes in tissues implicated in HMs are also highly expressed in non-HM-related tissues. However, *SRSF1* and *SMURF1* showed subtle differences in gene expression levels.

### Hub gene survival analysis

**Figure 8:**
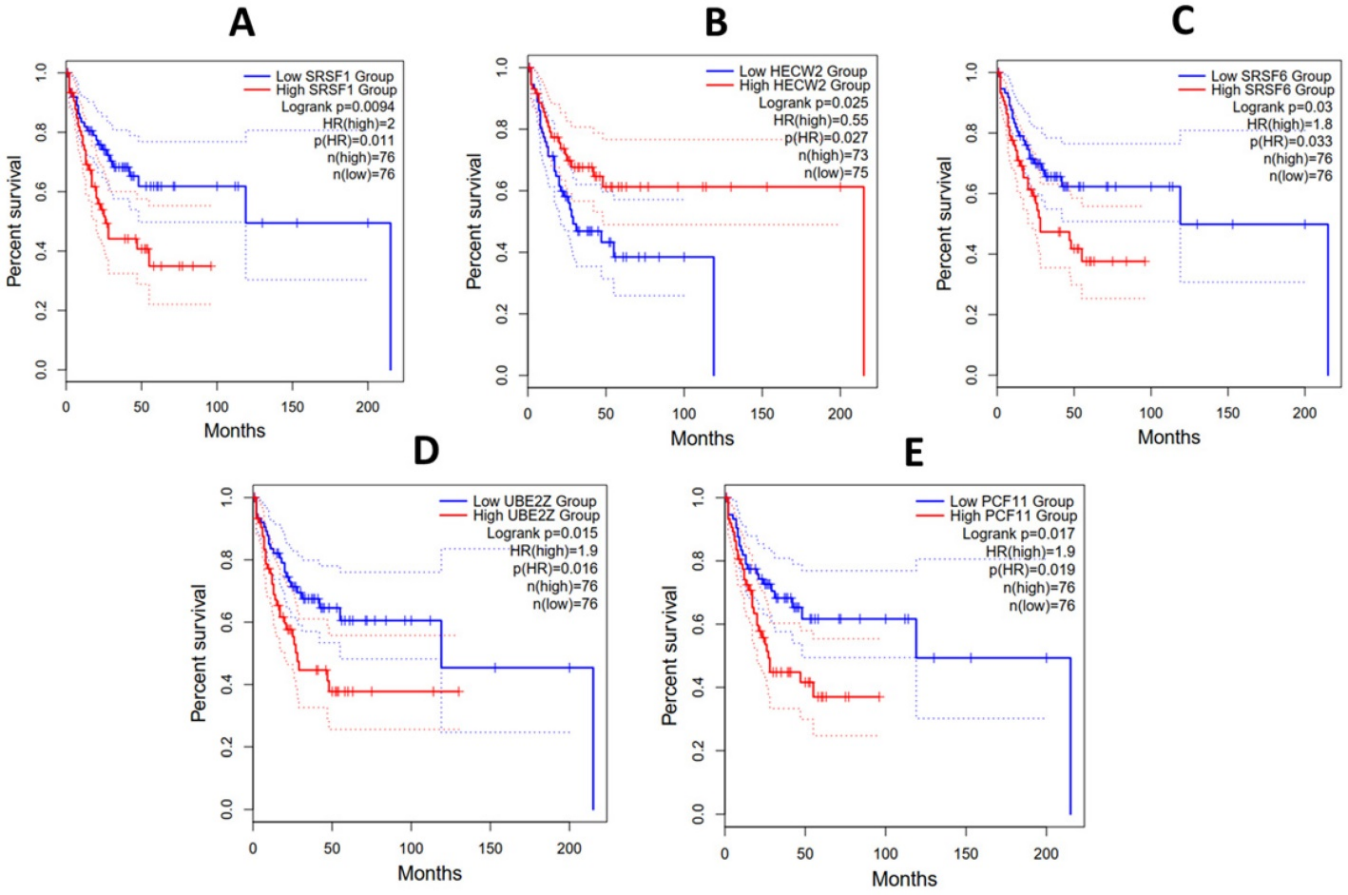
Overall survival analysis of (A) *SRSF1*, (B) *HECW2*, (C) *SRSF6*, (D) *UBE2Z* and (E) *PCF11* in patients with Acute Myeloid Leukemia and Diffuse Large B-cell Lymphoma from the TCGA project. The x and y axes represent the survival time in months and survival probability, respectively.

The prognostic role of all the hub genes unraveled was investigated using the Kaplan-Meier method. The survival plots were used to measure the length of time it takes an event to occur in different patient groups. Hub genes with associated P_adj_ values greater than or equal to 0.05 were excluded. Figure 8 shows that in DLBC and LAML, high *SRSF6*, *UBE2Z*, *PCF11* and *SRSF1* expression was associated with poor prognosis. Additionally, patients with low expression of *HECW2* exhibited a lower survival advantage than those with higher expression levels. While making these extrapolations, we considered the median survival proportions from the y-axis of Figure 8.

### Somatic copy number alterations (SCNA) and Tumor immune infiltration level (TIIL) analysis

**Figure 9:**
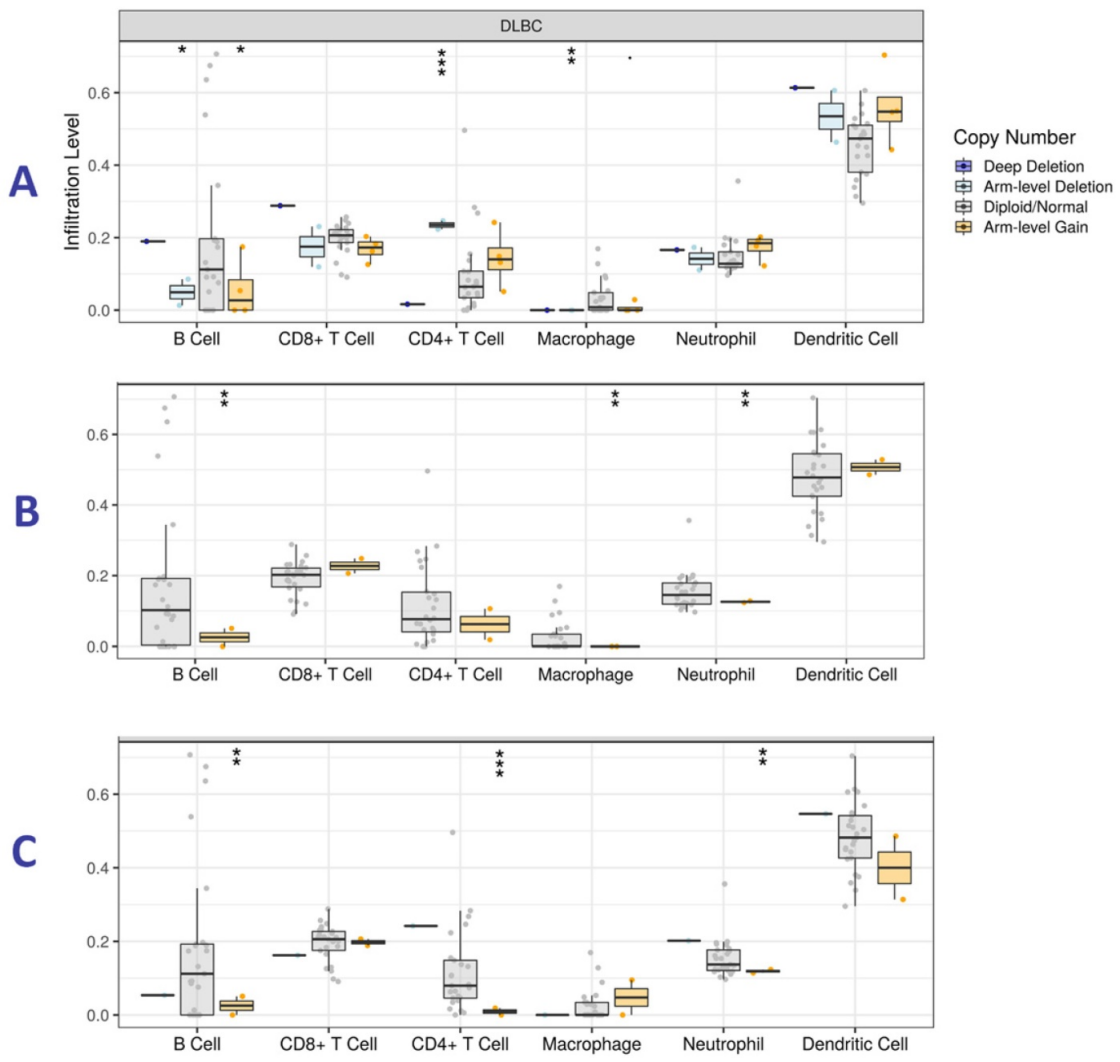
Tumor immune infiltration levels analysis for (A) *CDC5L*, (B) *HNRNPH1* and (C) *RBCK1* in Diffuse Large B-cell Lymphoma (DLBC). The y-axis represents infiltration levels. P value definitions: 0 ≤ *** < 0.001 ≤ ** < 0.01 ≤ * < 0.05 ≤. < 0.1.

TIMER online tool was used to determine the presence of SCNAs and tumor immune cells (TICs) in HM patients. We focused on DLBCL since it is the only hematologic malignancy available in TIMER. Statistical significance in associations was measured with the two-sided Wilcoxon rank-sum test while analyzing all the hub genes. Here, we report on genes with higher levels of statistical significance in the immune cells under study. High expression of *CDC5L*, *HNRNPH1* and *RBCK1* was associated with infiltration by TICs, especially B cells (Figure 9), indicating a possible association between the genes and immune response.

### Gene expression in patients of different age groups, races and gender

The UALCAN web tool was used to explore the difference in hub gene expression between races, age groups and gender of patients. *HNRNPH1* had a significant difference in expression in patients of different races, such as Caucasian vs. Asians (p=2.32×10^-2^) and African/American vs. Asians (p=6.84×10^-3^). However, there was no significant difference in expression between Caucasians and Africans/Americans. Additionally, *FBXO41* expression in patients of various age groups showed significant differences in the following pairs: 21-40 vs. 81-100 (p=9.82×10^-4^), 41-60 vs. 81-100 (p=5.57×10^-4^) and 61-80 vs. 81-100 (p=2.97×10^-4^).

## Discussion

Hematological malignancies mortality rate remains high and constitutes about 11.5% of all cancer cases worldwide. The poor prognosis could be attributed to a limited understanding of its pathogenesis and other underlying mechanisms of HMs. In the present study, RNA-seq data from four HMs were integrated and analyzed to establish a typical gene expression pattern and other biological mechanisms that could guide the development of novel diagnostics for early detection and treatment to improve the prognosis of HMs.

In total, 2136 genes were differentially expressed between the HMs and non-HM controls. Subsequent gene ontology and pathway enrichment analyses revealed the genes to be enriched in steroid and cholesterol biosynthesis, cell cycle regulation and regulation of *SREBF* expression. Cholesterol is a precursor to steroid hormones and bile acids, which play critical roles in cell growth and differentiation 39. In tumorigenesis and cancer progression, cholesterol can modulate signaling pathways by covalently binding to and modifying proteins such as hedgehog and smoothened 40, 41. These have been observed in colon cancer 42, breast cancer 43 and prostate cancer 44. *SREBF*, a master transcription factor, has also been reported to be upregulated in several human cancers, including glioblastoma 45. Overall, cholesterol metabolism plays a significant role in cancer metastasis, progression, proliferation and differentiation 46, 47. Investigating these critical pathways could help us better understand how HMs develop and may point to more reliable ways of diagnosis and treatment.

We created a PPI network for systematic analysis to investigate the pathogenesis of HMs. We avoided the introduction of noise and incomplete data that may affect the PPI network by setting the minimum interaction to 0.9 out of a possible 1.0. The resulting PPI network was run through MCODE, which used the connection data to find dense regions within the PPI networks. The network analysis revealed that there were 61 modules in the network, each with an accompanying score. The most closely connected module in the network was the first-rank module, which had a score of 29.54 and contained 60 genes. Studies Xia *et al.* 48, Yang *et al.* 49 and Yang *et al.* 50 on cervical cancer, glioblastoma and head and neck cancer, respectively, showed that modular analyses could be used to isolate related genes accurately and further accentuates the relevance of modular approach in the screening for biomarkers. The genes in the module with the highest scores were the ones that influenced HM occurrence.

Next, we performed hub gene co-expression analysis using STRING to confirm the interactions between the genes. Notably, we found *SRSF1*, *HECW2*, *SRSF6*, *UBE2Z* and *PCF11,* to be linked to carcinogenesis and cancer management 51-62 and are associated with poor prognosis in HMs. We also found that a high level of expression of *PCF11* is associated with poor prognosis in HM. This is consistent with findings from Ogorodnikov *et al.* 63, in which low expression of *PCF11* was associated with a good prognosis in neuroblastoma. The exact role of *PCF11* in cancer development and progressing remains to be determined. However, evidence implicates *PCF11* in cancers, including head and neck squamous cell carcinoma 64 and oral squamous cell carcinoma 65.

The *PCF11* (Cleavage and Polyadenylation Factor Subunit) gene product is an mRNA 3' end processing complex protein, which plays a crucial role in producing mRNA isoforms with varying 3' untranslated region (UTR) lengths. 3' UTRs shortening is a hallmark of most cancer cells and that ubiquitination of *PCF11* through MAGE-A11-HUWE1 ubiquitin ligase promotes 3' UTRs shortening that drives tumorigenesis 66.

Interestingly, we found *HECW2* to be downregulated. E3 Ubiquitin-Protein Ligase gene (*HECW2*) codes for a member of the E3 ubiquitin ligase family and has been demonstrated to play a significant role in angiogenesis, the process by which new capillaries form from pre-existing blood vessels 67. Many solid tumors, including HMs, require angiogenesis for growth and metastasis. *HECW2* stabilizes *AMOTL1*, a cell-to-cell junction regulator; knockout of *HECW2* in endothelial cells increases the rate of vascular permeability and sprouting angiogenesis 67. Angiogenesis inhibition is a well-established treatment approach for many solid cancers. The anti-angiogenic role of *HECW2* could be further explored as a potential therapeutic target.

Ubiquitin Conjugating Enzyme E2 Z (*UBE2Z*) is involved in the degradation of defective proteins and has been shown to be highly expressed in hepatocellular carcinoma compared to healthy controls and results in poor prognosis 68. Gene knockout analysis of *UBE2Z* using siRNA has been found to drastically reduce tumor cell proliferation, migration and invasion 68. These findings suggest that *UBE2Z* could be a predictive biomarker for human cancer, including hematological malignancies.

Alternative splicing (AS) is found in nearly every human gene, and aberrant alternative splicing has been associated with cancer 66. The archetypal member of the serine/arginine-rich protein family, *SRSF6*, a proto-oncogene, has been identified as a significant regulator of alternative splicing in cancer-associated genes 69. *SRSF6* has been demonstrated to contribute to the regulation of alternative splicing in cervical cancer patients 66. Studies by Yang *et al*. 66 revealed that in comparison to control cells, *SRSF6* overexpression resulted in significantly increased apoptosis and decreased cell proliferation. Transcriptome analysis also showed that overexpression of *SRSF6* in cancer cells induced large-scale changes in transcriptional expression levels and alternative splicing.

Additionally, AS genes have been implicated in DNA damage response (DDR) pathways such as double-strand break repair. Yang *et al*.'s report indicate that *SRSF6* can influence cancer growth by activating DDR pathways via AS regulation. These findings add to our understanding of the mechanisms behind *SRSF6*-mediated gene regulation and points to the possibility of using *SRSF6* as a cancer therapeutic target. *SRSF6* is also highly expressed in skin cancer 70, pancreatic cancer 71, breast cancer 72 and colorectal cancer 73 and promotes the survival of cancer cells. *SRSF6* has also been found to regulate exon skipping, making it highly important in the survival of leukemic cells 74. Moreover, Moradpoor *et al*. 75 used *SRSF6* to distinguish between metastatic and non-metastatic breast cancer at the time of diagnosis.

*SRSF1* also belongs to the arginine/serine splicing factor family of genes, preventing exon skipping, invasion, and senescence and regulating splicing activities 76. Dong *et al*. 77 found that downregulation of *SRSF1* was associated with reduced apoptosis, proliferation and metastasis in cervical cancer patients. Zhou *et al.* 76 reported *SRSF1* as a major onco-driver in several human cancers, including gastric cancer. Its overexpression has been linked to increased cell proliferation and metastasis of cancer cells, making it a potential candidate for further research as a prognostic biomarker in hematological malignancies. *SRSF1* is consistently overexpressed in breast cancer samples and positively correlates with tumor grade and poor prognosis 78. It also has the potential of increasing the rate of cell proliferation, migration and inhibition of apoptosis. Studies by Lei *et al*. 79 revealed that *SRSF1* promoted tumor cell invasion and metastasis in hepatocellular carcinoma. Additionally, the knockout of *SRSF1* in mouse models resulted in the inhibition of tumor cell migration.

To sum up,* SRSF1*, *HECW2*, *SRSF6*, *UBE2Z* and *PCF11* are implicated in the proliferation, apoptosis or metastasis of cancer cells and offer potential research avenues for use as diagnostic and prognostic biomarkers of HM management.

## Conclusion

The present study compared HM gene expression patterns to non-HM samples and revealed five genes,* SRSF1*, *HECW2*, *SRSF6*, *UBE2Z* and *PCF11* to be associated with poor prognosis of HMs. The genes are novel and their exact contribution to HMs development and progression is unclear. Further research is needed to understand the precise mechanism by which gene deregulation leads to poor prognosis in HMs. The findings also provide important clues for HMs and could serve as prognostic markers for HM treatment and management.

## Supplementary Material

Supplementary table.

## Figures and Tables

**Figure 10:**
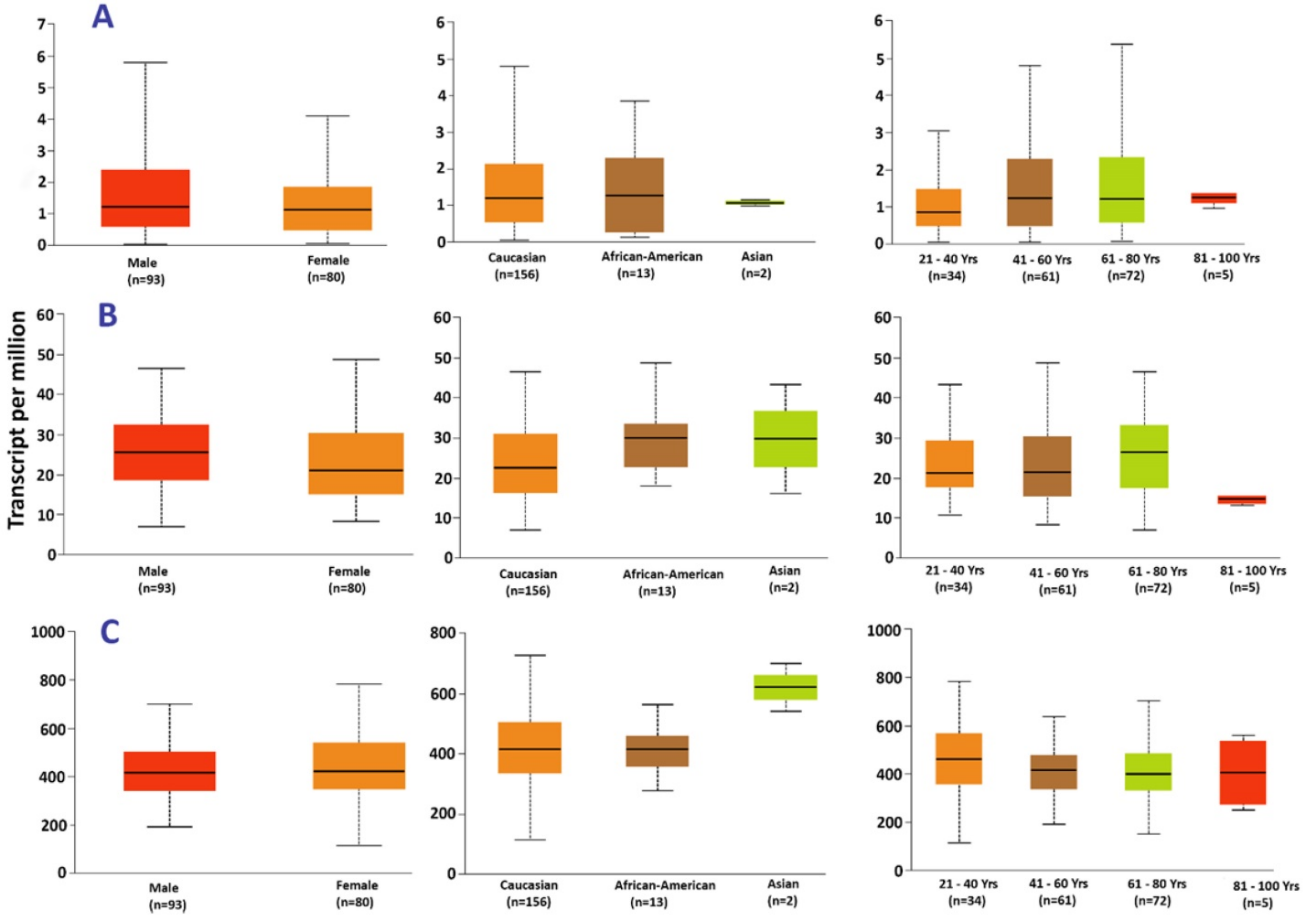
Expression of (A) *ASB2*, (B) *FXB041*, and (C) *HNRNPH1* in acute myeloid leukemia based on patient's gender, race and age.

**Table 1:** Characteristics of the RNA-seq dataset used in this study

Data accession	Contributors	Organism	Year	Cancer type	Number of samples
PRJNA594725	CNAG-CRG 18	Homo sapiens	2019	CLL	10
PRJNA528267	Cocciardi *et al*. 15	Homo sapiens	2019	AML	10
PRJNA475681	Black *et al*. 16	Homo sapiens	2018	ALL	10
SRP099346	Lombardo *et al*. 17	Homo sapiens	2017	BL	10
ERP001942	Ouyang *et al.* 19	Homo sapiens	2017	LCLs	10

**Table 2:** Characteristics of all tools used before differential expression analysis in R

Tool	Version	Function	Reference
FastQC	0.11.9	Quality checks	Andrews 20
MultiQC	1.10	Summarization	Ewels *et al.* 21
Trimmomatic	0.39	Trimming	Bolger *et al.* 22
STAR	2.7.7a	Splice-aware alignment	Dobin *et al.* 23
featureCounts	1.6.3	Gene quantification	Liao *et al.* 24

**Table 3:** Characteristics of the tools used for differential expression analysis

DEA tool	Version	Read count distribution	Normalization approach	Differential expression test	Citation
DESeq2	1.28.1	Negative binomial	size factors	Exact test	Love *et al.* 25
edgeR	3.30.3	Negative binomial	trimmed mean of M-values (TMM)	Exact test	Robinson *et al.* 26
limma+voom	3.44.3	voom transformation of counts	trimmed mean of M-values (TMM)	Empirical Bayes method	Ritchie *et al.* 27

**Table 4:** Web-based tools used in the analysis of differentially expressed genes

Tool	Function	Reference
DAVID	Gene enrichment analysis	Huang *et al.* 28
G:profiler	Gene enrichment analysis	Reimand *et al.* 29
Cytoscape	PPI network	Shannon *et al.* 30
STRING	PPI network	Szklarczyk *et al.* 31
GEPIA	Survival analysis	Tang *et al.* 33
TIMER	Tumor immune infiltration detection	Li *et al.* 34
UALCAN	Gene expression in different races, gender and age groups	Chandrashekar *et al.* 37

**Table 5:** Gene and pathway enrichment analysis of the common DEGs

Term ID	Term description	Number of genes	P_adj_
**Molecular function**			
GO:0005515	Protein binding	1695	3.308×10^-18^
GO:0003824	Catalytic activity	696	2.158×10^-10^
GO:0042802	Identical protein binding	289	1.395×10^-7^
GO:0016740	Transferase activity	309	1.627×10^-6^
GO:0019899	Enzyme binding	275	1.911×10^-5^
**Biological process**			
GO:0006996	Organelle organization	528	5.599×10^-10^
GO:0071840	Cellular component organization or biogenesis	782	5.929×10^-8^
GO:0044237	Cellular metabolic process	1222	6.526×10^-8^
GO:0008152	Metabolic process	1299	3.326×10^-7^
GO:1902531	Regulation of intracellular signal transduction	258	3.855×10^-7^
**Cellular component**			
GO:0005622	Intracellular anatomical structure	1733	1.4×10^-46^
GO:0005737	Cytoplasm	1413	5.435×10^-39^
GO:0005829	Cytosol	777	5.924×10^-30^
GO:0043227	Membrane-bounded organelle	1491	1.869×10^-28^
GO:0043229	Intracellular organelle	1455	8.918×10^-27^
**KEGG Pathway**			
KEGG:00100	Steroid biosynthesis	11	5.075×10^-4^
KEGG:01100	Metabolic pathways	226	2.443×10^-3^
KEGG:01200	Carbon metabolism	30	3.282×10^-3^
KEGG:00010	Glycolysis / Gluconeogenesis	19	2.702×10^-2^
KEGG:00620	Pyruvate metabolism	15	2.999×10^-2^
**Reactome Pathway**			
REAC:R-HSA-1655829	Cholesterol biosynthesis	17	2.525×10^-8^
REAC:R-HSA-191273	Cell Cycle, Mitotic	108	3.632×10^-6^
REAC:R-HSA-69278	Regulation of cholesterol biosynthesis by SREBP	23	8.498×10^-6^
REAC:R-HSA-2426168	Activation of gene expression by SREBF	18	1.617×10^-4^
REAC:R-HSA-5419276	Mitochondrial translation termination	28	4.817×10^-4^
**Human phenotype**			
HP:0000252	Microcephaly	182	3.154×10^-3^
HP:0002977	Aplasia/Hypoplasia involving the central nervous system	239	3.624×10^-3^
HP:0040195	Decreased head circumference	182	7.637×10^-3^
HP:0004377	Hematologic neoplasm	53	1.027×10^-2^
HP:0011893	Abnormal leukocyte count	71	1.051×10^-2^
HP:0010975	Abnormal B cell count	15	1.264×10^-2^
HP:0001882	Leukopenia	49	1.407×10^-2^
HP:0002846	Abnormal B cell morphology	15	1.892×10^-2^

## Abbreviations

HMs: Hematologic malignancies
TCGA: The Cancer Genome Atlas
CPTAC: Clinical Proteomic Tumor Analysis Consortium
GTEx: Genotype-Tissue Expression
RNA-seq: RNA sequencing
DALYs: Disability-adjusted life-years
NHL: Non-Hodgkin's lymphoma
CLL: Chronic lymphocytic leukemia
AML: Acute myeloid leukemia
ALL: Acute lymphocytic leukemia
BL: Burkitt lymphoma
LCLs: lymphoblastoid cell lines
CNAG-CRG: National Center for Genomic Analysis-Centre for Genomic Regulation
DEA: Differential expression analysis
TMM: trimmed mean of M-values
GO: Gene ontology
DAVID: Database for Annotation, Visualization and Integrated Discovery
KEGG: Kyoto Encyclopedia of Genes and Genomes
DEGs: differentially expressed genes
MCODE: Molecular Complex Detection
PPI: Protein-protein interaction network
DLBC: Diffuse Large B-cell Lymphoma
TIMER: Tumor Immune Estimation Resource
GEPIA: Gene Expression Profiling Interactive Analysis
TIIC: tumor infiltration immune cells
SCNAs: somatic copy number alterations
STRING: search tool for retrieval of interacting genes
