# Supplementary material for: New Clues to Prognostic Biomarkers of Four Hematological Malignancies
Source: J Cancer. 2022 May 9;13(8):2490–503. doi: 10.7150/jca.69274 (PMC9174851; doi:10.7150/jca.69274)
Supplement: Supplementary file 1 — Supplementary table. [file jcav13p2490s1.pdf]

Supplementary Table S1: Gene and pathway enrichment analysis of the sixty hub genes

**Term ID**	**Term description**	**Number of genes**	P_{adj}
**Molecular function**			
GO:0004842	Ubiquitin-protein transferase activity	20	3.78×10^{-16}
GO:0019787	Ubiquitin-like protein transferase activity	20	1.08×10^{-15}
GO:0003723	RNA binding	28	8.38×10^{-11}
GO:0061630	Ubiquitin protein ligase activity	11	5.08×10^{-07}
GO:0061659	Ubiquitin-like protein ligase activity	11	7.77×10^{-07}
GO:0140096	Catalytic activity, acting on a protein	24	3.76×10^{-06}
**Biological process**			
GO:0000377	RNA splicing, via transesterification reactions	32	1.55×10^{-35}
GO:0000398	mRNA splicing, via spliceosome	32	1.55×10^{-35}
GO:0000375	RNA splicing, via transesterification reactions	32	2.06×10^{-35}
GO:0008380	RNA splicing	32	6.21×10^{-33}
GO:0006397	mRNA processing	32	4.88×10^{-32}
GO:0016071	mRNA metabolic process	32	4.21×10^{-24}
**Cellular component**			
GO:0005681	Spliceosomal complex	18	4.14×10^{-20}
GO:0071013	Catalytic step 2 spliceosome	14	4.32×10^{-18}
GO:0005684	U2-type spliceosomal complex	14	1.41×10^{-17}
GO:1902494	Catalytic complex	29	2.24×10^{-15}
GO:0097525	Spliceosomal snRNP complex	11	1.66×10^{-14}
GO:0030532	Small nuclear ribonucleoprotein complex	11	8.91×10^{-14}
**KEGG Pathway**			
KEGG:03040	Spliceosome	20	1.44×10^{-23}
KEGG:03015	mRNA surveillance pathway	7	2.24×10^{-05}
KEGG:04120	Ubiquitin mediated proteolysis	5	0.033
